# Examining the utility of the CD64 index compared with other conventional indices for early diagnosis of neonatal infection

**DOI:** 10.1038/s41598-018-28352-7

**Published:** 2018-07-03

**Authors:** Zongsheng Tang, Daojian Qin, Mingfen Tao, Kun Lv, Shuli Chen, Xiaolong Zhu, Xueqin Li, Tianbing Chen, Mengying Zhang, Min Zhong, Hui Yang, Yang Xu, Shuanggen Mao

**Affiliations:** 1 Central Laboratory, Yijishan Hospital, Wannan Medical College, Wuhu, 241001 Anhui China; 2 Department of Pediatrics, Yijishan Hospital, Wannan Medical College, Wuhu, 241001 Anhui China; 3 Hemopurification Center, Yijishan Hospital, Wannan Medical College, Wuhu, 241001 Anhui China

## Abstract

As specific clinical manifestations and detection tools for early neonatal infections are lacking, early detection and treatment are ongoing challenges. The present study aimed to investigate the role and clinical significance of the CD64 index in comparison with conventional examination indices (WBC, PCT and CRP) for the early diagnosis of neonatal infection. Of 74 in-patient newborns, non-sepsis (non-specific infection but free of sepsis), sepsis and control [newborns with ABO hemolytic disease of the newborn (ABOHDN) but without infection] groups involved 32, 16 and 26 cases, respectively. Peripheral blood WBC, PCT, CRP and CD64 indices were acquired for all groups. The sepsis group showed significantly higher WBC, PCT and CRP levels than the control group. Compared with the non-sepsis group, the sepsis group demonstrated significant increases in PCT but not in WBC or CRP. Compared with the control group, the non-sepsis and sepsis groups had higher CD64 indices. Combined, compared with the WBC, PCT and CRP indices, the CD64 index is unique in its capacity to diagnose neonatal infections early. The CD64 index combined with other conventional indices may lay a basis for the future early diagnosis and effective treatment of neonatal infections.

## Introduction

Neonatal sepsis has become a global health problem due to its high morbidity and mortality^1^. Worldwide, approximately 0.6 million newborns die from sepsis each year, which accounts for 22% of neonatal deaths. This is a serious challenge for clinicians^2,3^. The diagnostic criterion for sepsis is blood culture, which usually takes 42–72 hours to obtain a result and has a low sensitivity^4,5^. These shortcomings often result in delayed and missed diagnoses. As neonatal sepsis is an advanced stage of non-sepsis, early diagnosis and treatment of neonatal infection is an important guarantee to prevent sepsis caused by non-sepsis.

However, compared with normal newborns, there is no specific clinical manifestation or test index for non-sepsis in newborns. At present, the indicators commonly used in the clinic are white blood cell (WBC), procalcitonin (PCT) and C-reactive protein (CRP) levels, which play important roles in the diagnosis of neonatal infection. The accuracy of WBC counts in distinguishing between infectious diseases and non-infectious inflammatory disorders is very low^6^. CRP is a well-studied acute phase protein that is synthesized and secreted in the liver. CRP is increased 24–48 hours after bacterial infection and belongs to the late-onset infection index^7,8^. CRP can assess the extent of infection and monitor antibiotic treatment^9,10^. However, the level of CRP is easily affected by many factors. CRP is physiologically elevated 3 days after birth and is influenced by gestational age, birth weight, rupture time of the fetal membranes, mode of delivery, perinatal non-infectious complications and the mother^8,11,12^.These effects can easily lead to clinical misdiagnosis, which can lead to the misuse of antibiotics. In addition, since the CRP level increases relatively late, it is not a timely reflection of the clinical status of neonatal infection. Treatment time is easy to mismanage, and the infection can be aggravated, leading to neonatal sepsis. Therefore, the value of CRP in the diagnosis of neonatal infection and sepsis should be reconsidered.

PCT is also an acute phase protein that is mainly synthesized and secreted by thyroid follicular cells under physiological conditions. Upon bacterial infection, bacterial endotoxin is released to induce inflammation. Procalcitonin begins to increase within 3–4 hours of infection, peaks at 6–8 hours and lasts for at least 24 hours^8,12,13^. However, the disadvantage of PCT is that procalcitonin can also increase physiologically within 48 hours after the birth of a healthy newborn, which increases the difficulty of diagnosis^12,14^. Similar to CRP, PCT is increased in some non-infectious perinatal complications, such as neonatal respiratory distress syndrome, perinatal asphyxia, intracranial hemorrhage, circulatory failure and cardiopulmonary resuscitation^15–17^. In addition, the use of antibiotics prenatally or perinatally in pregnant women or in neonates postpartum can also affect the level of PCT^18^. Due to the low sensitivity or specificity of WBC, CRP and PCT, it is easy to misdiagnose or miss the diagnosis of neonatal infection during clinical diagnosis and treatment. Therefore, it is of great significance to find a new biomarker for the early diagnosis of neonatal bacterial infection and to carry out objective clinical evaluations.

Many studies have shown that CD64 molecules can be used as biomarkers for the diagnosis of bacterial infection and sepsis. CD64, also known as Fc-gamma receptor 1 (FcγR1), is mainly distributed on the surface of macrophages, dendritic cells and monocytes, with almost no expression on neutrophils under physiological conditions^9,19,20^. Upon bacterial infection or inflammatory damage, CD64 is highly expressed on the surface of neutrophils and is elevated to 5–10 times the normal level within 1–6 hours^21–23^. CD64 can be used as an indicator in the early diagnosis of neonatal infections as it has no physiological changes and is unaffected during the perinatal period or by complications, gestational age, and its expression is similar in normal preterm infants, full-term infants, babies and adults^9,11,24,25^. With the development of flow cytometry, CD64 can be measured quickly and accurately. This measurement, which only uses a small amount of whole blood cells, avoids iatrogenic anemia. Therefore, it is convenient for the early diagnosis and treatment of neonatal infections.

However, previous studies have produced conflicting findings regarding the sensitivity, specificity and cutoff of CD64 for the early diagnosis of neonatal infection. The current study aims to compare the differences in the CD64 index [the granulocyte:lymphocyte ratio of CD64 expression via mean fluorescence intensity (MFI)] and conventional clinical indicators (WBC, PCT and CRP) among the control, non-sepsis and sepsis groups by analyzing the receiver-operating characteristic curves (ROC), thus determining their value in clinical practice in the early diagnosis of neonatal infection.

## Materials and Methods

### Study subjects

All 74 cases were from the neonatal intensive care unit of Yijishan Hospital, Wannan Medical College, China. Among them, there were 46 males and 28 females, and the duration of the illness in each case was less than 3 days. According to the disease, the children were divided into three groups: non-sepsis (n = 32), sepsis (n = 16) and control (n = 26). The inclusion criteria for the non-sepsis group were common non-specific infections, whereby at least one WBC, PCT or CRP test result was abnormal but did not meet the clinical diagnostic criteria for sepsis. Non-specific infections included neonatal omphalitis, local infection, pneumonia, enteritis and/or demonstrating infection symptoms, such as abdominal distension, pathological jaundice, skin color change and hepatosplenomegaly. The sepsis group included patients with positive blood cultures and patients with negative blood cultures who were clinically diagnosed with sepsis. The diagnostic criteria for sepsis were based on the standard of the 8th edition of Pediatrics^26^. The neonates with ABOHDN, but without infection, were selected as the control group based on clinical manifestations and auxiliary examinations. The exclusion criteria for all subjects were neonatal asphyxia, neonatal respiratory distress syndrome, fetal fecal aspiration syndrome, pulmonary hemorrhage, congenital deformity and antibiotic treatment before admission. All subjects were examined for routine blood tests, including the CRP, PCT and CD64 indices; blood culture; and other indicators before using antibiotics after admission. The study was approved by the Medical Ethics Committee of Yijishan Hospital at Wannan Medical College, China. All methods were performed in accordance with the relevant guidelines and regulations. After admission, the families of the children were notified of all the tests of the indicators of infection and provided written informed consent before blood collection.

### Flow cytometric detection of CD64 MFI and calculation of the CD64 index

Blood samples were collected in tubes with EDTA anticoagulant. Whole blood was stained with anti-human CD45 phycoerythrin-cyanine5 (CD45-PE-Cy5; eBioscience, USA) and anti-human CD64 phycoerythrin (CD64-PE; Beckman Coulter, USA). After staining, red blood cells were lysed with Erythrocyte Lysis Buffer (BD Biosciences, USA) to obtain peripheral blood mononuclear cells (PBMC). PBMC stained with fluorescent antibodies were examined by flow cytometry (Cytomics™ FC 500, Beckman Coulter, USA). The data were analyzed by flow cytometry analysis software (FlowJo 7.6, LLC, USA). The scatter plot was drawn with side scatter (SSC) and CD45-PE-Cy5, and neutrophils and lymphocytes were gated to acquire their CD64 MFI. The CD64 index was determined by the ratio of the CD64 MFI of granulocytes to that of lymphocytes.

### The examination of clinical infection indicators: WBC, PCT and CRP

The PCT, CRP and WBC levels in the peripheral blood of patients were analyzed at a clinical laboratory in Yijishan Hospital, Wannan Medical College. The PCT was tested using a chemiluminescent immunoassay with an automatic chemiluminescence apparatus (Snibe Diagnostic MAGLUMI 1000) and diagnostic kits (both provided by Shenzhen New Industries Biomedical Engineering Co., Ltd, China). The CRP was tested using a particle-enhanced turbidimetric immunoassay with a HITACHI 7600 Series Automatic Biochemical Analyzer (Hitachi, Ltd., Japan) and diagnostic kits (DiaSys Diagnostic Systems GmbH, Shanghai, China). Routine blood counts were performed with a Mindray BC-6900 hematology analyzer (Mindray Bio-Medical Electronics Co., Ltd., Shenzhen, China). The WBC and neutrophil/lymphocyte ratio (NLR) were calculated based on the routine blood count results. All operations were strictly conducted according to the instructions provided by the instrument and reagent manufacturers.

### Statistical analysis

GraphPad Prism (version 6.0) statistical software was used for the data analysis. An inter-group comparison was performed by analysis of variance. The correlations between the different parameters were completed using a Spearman’s test. The sensitivity and specificity of the different methods were compared by analyzing the area under the ROC curve. Categorical variables were presented as absolute number and percentage, and were compared using Chi-square test. The differences between groups were statistically significant if the statistical results showed P < 0.05.

## Results

### The Details Of Study Population according to groups

A total of 74 neonates were involved in this study. Subjects were divided into three groups: non-sepsis (n = 32; 21 males and 11 females), sepsis (n = 16; 10 males and 6 females) and control (n = 26; 15 males and 11 females). Demographic characteristics of the three groups were presented in Table 1. The table showed that there were no significant differences in gender, birth weight, gestational age, postnatal age and delivery mode among the three groups (P = 0.8250, P = 0.3953, P = 0.4401, P = 0.3951 and P = 0.7685 respectively).

**Table 1:** Characteristics of neonatal population according to groups.

Variables	Non-sepsis (n = 32)	Sepsis (n = 16)	Control (n = 26)	p-values
Male gender, n (%)	21 (65.6)	10 (62.5)	15 (57.7)	0.8250^a^
Birth weight (g)	3194 ± 309	3136 ± 153	3092 ± 307	0.3953^b^
Gestational age (weeks)	38.65 ± 0.81	38.93 ± 0.86	38.88 ± 0.83	0.4401^b^
Postnatal age (days)	3.69 ± 1.12	3.28 ± 0.94	3.47 ± 0.91	0.3951^b^
Vaginal delivery, n (%)	14 (43.75)	6 (37.5)	9 (34.62)	0.7685^a^

^a^Analyzed by Chi-square test. ^b^Analyzed by ANOVA test; Values are presented as mean with standard deviation (SD) or number (%).

### Differences in conventional infection indicators: WBC, PCT and CRP in the control, non-sepsis and sepsis groups

**Figure 1:**
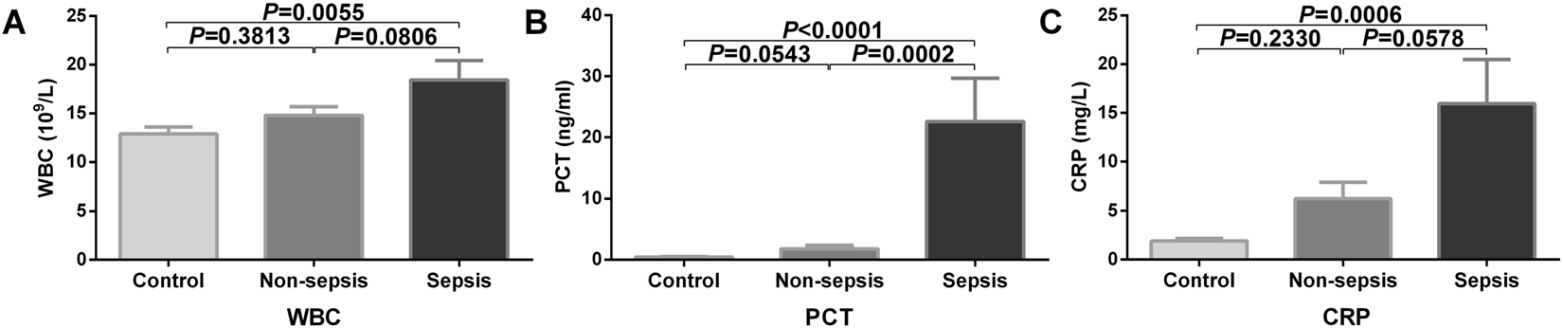
Differences in the WBC, PCT and CRP levels between groups. (**A**) Comparison of the differences in WBC among the control, non-sepsis and sepsis groups. (**B**) Comparison of the differences in PCT among the control, non-sepsis and sepsis groups. (**C**) Comparison of the differences in CRP among the control, non-sepsis and sepsis groups.

Neonatal infections tend to cause an increase in one or more of the WBC, PCT, and CRP indicators. To reflect the value of these parameters in neonatal infection, this study compared their differences among different groups. The study found that the WBC in the sepsis group was significantly higher than that in the normal control group, but no differences were observed in WBC between the sepsis group and the non-sepsis group or between the non-sepsis group and the normal control group (P = 0.0055, P = 0.0806 and P = 0.3813 respectively; Fig. 1A). However, the PCT parameters in the sepsis group were statistically higher than those in the non-sepsis and control groups, and significant differences were not observed between the non-sepsis group and the control group (P = 0.0002, P < 0.0001 and P = 0.0543 respectively; Fig. 1B). Furthermore, the CRP parameters in the sepsis group were statistically higher than in the control group but did not show significant differences between the non-sepsis and sepsis groups or the control group (P = 0.0006, P = 0.0578 and P = 0.2330 respectively; Fig. 1C).

### Different levels of the CD64 index and correlations with WBC, PCT and CRP in the control, non-sepsis and sepsis groups

**Figure 2:**
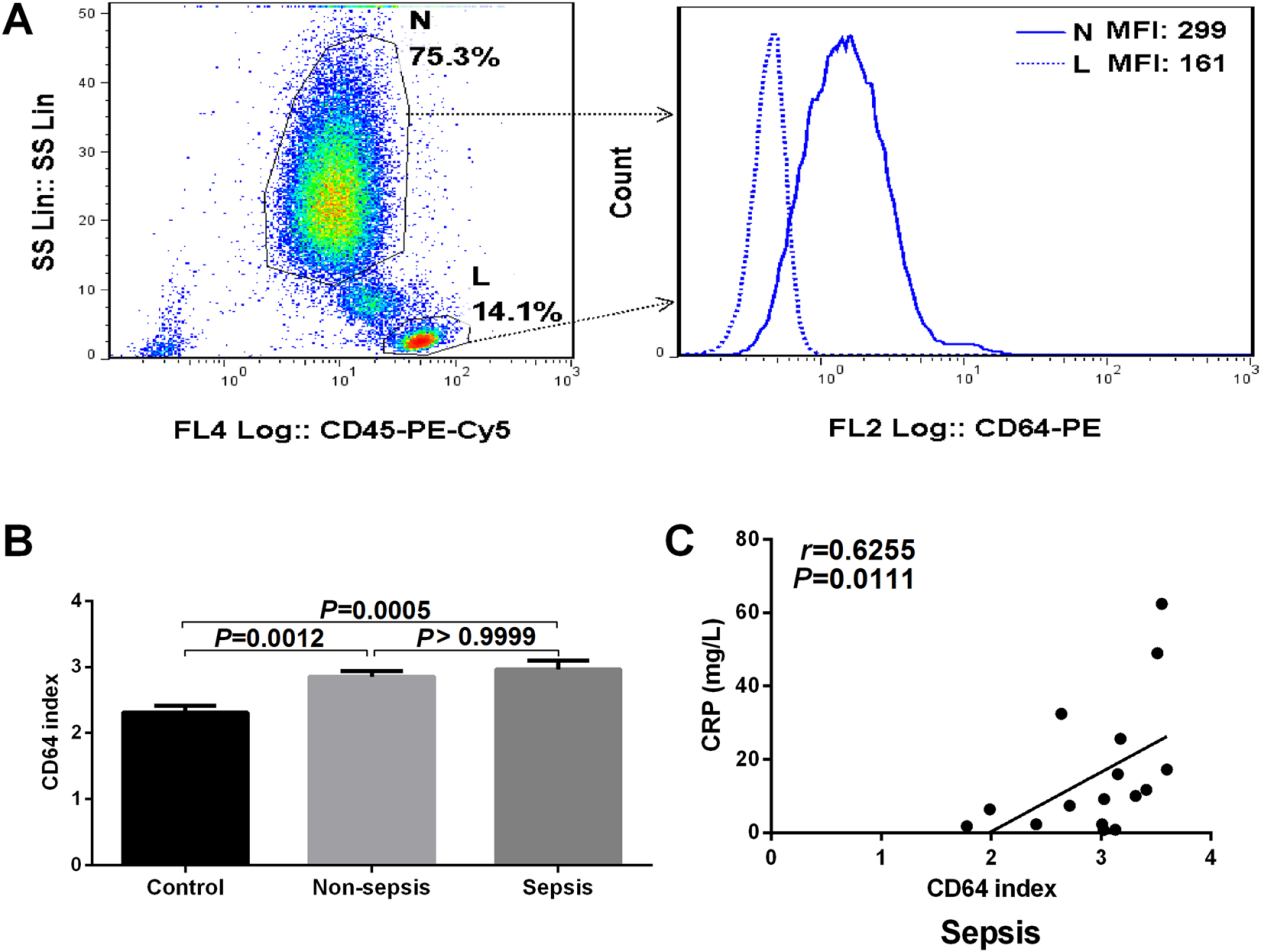
The CD64 index and correlations with conventional infection indicators. (**A**) Representative flow cytometric scatter plots with gating strategy and histogram showing the percentage (left) and MFI (right) of neutrophils (N) and lymphocytes (L). (**B**) The CD64 index showed higher levels in the non-sepsis group and the sepsis group than in the control group. (**C**) The CD64 index had a positive correlation with CRP in the sepsis group.

In this study, to consider the experimental errors caused by the background levels of CD64 expression on neutrophils, the CD64 index was adopted as a diagnostic marker of infection. Representative flow cytometric scatter plots of SSC vs. CD45-PE-Cy5 with a gating strategy for neutrophils (N) and lymphocytes (L) are demonstrated in Fig. 2A (left). The flow cytometric histograms of the CD64 count and CD64 MFI on N and L derived from the scatter plot are shown in Fig. 2A (right). The CD64 index was from the MFI of CD64 on N and L. We found that the levels of the CD64 index both in the non-sepsis group and in the sepsis group were significantly higher than that in the control group, but there were no differences between the non-sepsis and sepsis groups (P = 0.0012, P = 0.0005 and P > 0.9999 respectively; Fig. 2B). In addition, through correlation analysis, this study found that there was a positive correlation between the CD64 index and CRP in the sepsis group (r = 0.6255, P = 0.0111; Fig. 2C).

### Correlation analysis of the CD64 index and NLR

**Figure 3:**
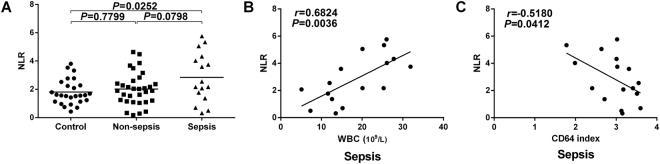
The difference in NLR between groups and the correlation with other infection indicators. (**A**) There was a markedly elevated NLR in the sepsis group compared with the control group. (**B**) In the sepsis group, NLR was positively correlated with WBC. (**C**) In the sepsis group, NLR was negatively correlated with the CD64 index.

NLR is an important subclass in the analysis of white blood cells; it contains two types of white blood cell subtypes and has a higher predictive value than any single index. NLR is mainly reported in tumors, appendicitis and post-operation, but there are few reports on neonatal sepsis and neonatal infection. Our study showed that there was a statistically significant difference in the NLR between the sepsis group and the control group, but no differences were observed between the sepsis and non-sepsis groups or between the non-sepsis and control groups (P = 0.0252, P = 0.0798 and P = 0.799; respectively; Fig. 3A). In addition, we found that in the sepsis group, NLR was positively correlated with WBC (r = 0.6824, P = 0.0036; Fig. 3B) but was negatively correlated with the CD64 index (r = −0.5180, P = 0.0412; Fig. 3C).

### The ROC curves of different biological indicators in the infection group (non-sepsis and sepsis; n = 48)

**Figure 4:**
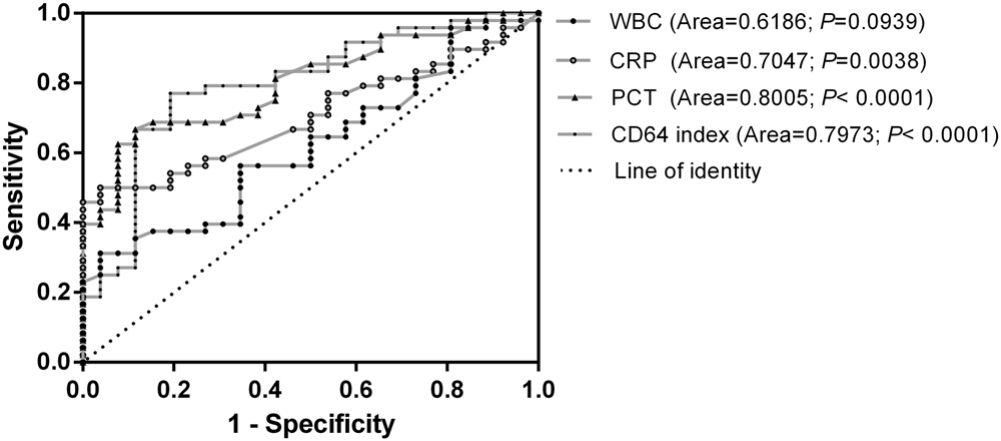
The ROC curves of CD64, PCT, CRP and WBC in the infection group. The ROC curves of CD64, PCT, CRP and WBC are represented by different lines.

By comparing the ROC curves of the CD64, PCT, CRP and WBC levels in the infected group, we found that the area under the curve (AUC) of the PCT (Area = 0.8005, P < 0.0001) was the largest and was slightly higher than that of the CD64 index (Area = 0.7973, P < 0.0001); the AUC of CRP (Area = 0.7047, P = 0.0038) was less than that of CD64 or PCT; and the AUC of WBC (Area = 0.6186, P = 0.0939) was the smallest and had no statistical significance (Fig. 4A).

## Discussion

The immune systems of newborns are immature and susceptible to pathogenic microorganisms. If the infection is not controlled promptly and efficiently, it can further develop into sepsis and be life threatening in severe cases. After infection, newborns can show non-specific clinical manifestations, such as crying, decreased movement, hypothermia, loss of appetite, not eating and weight loss, making the early detection of infection difficult. In the clinic, using antibiotics for non-infectious inflammatory or viral infections could result in an array of problems, such as prolonged hospitalization, increased treatment costs, adverse drug reactions and antibiotic resistanc^6,27,28^. Therefore, infected and septicemic newborns should be diagnosed accurately and treated as soon as possible.

However, at present, the detection methods for neonatal infection in China are more traditional, such as WBC, PCT and CRP. These traditional inspection items have different clinical significances at different stages of infection. The blind selection of single-item examination might lead to false negative results, causing missed diagnoses. Some scholars, after performing similar studies in other countries, believe that the CD64, PCT and CRP indices should be tested in the early (2–12 hours), middle (12–24 hours) and late (more than 24 hours) stages of infection, respectively^12^. Lam *et al*.^29^ monitored the CD64 index daily for necrotizing enterocolitis and sepsis in neonates and found that CD64 increased 1.5 days before the onset of infection. These data suggested that CD64 might be an important biomarker for the early diagnosis of infection. In our study, we sought to examine the early detection, diagnosis and early treatment of neonatal infections with nonspecific symptoms and physical signs. We found that the traditional biomarkers of WBC, CRP and PCT in the study of 43 cases of neonatal infection showed significant differences between the sepsis group and normal control group. Clinically, these traditional indicators have a relatively clear negative predictive value, which indicates that the traditional indicators are useful. However, their positive predictive value for sepsis is not satisfactory^30^. In this study, the traditional biomarkers could not distinguish between the non-sepsis group and the control group, which, in clinical work, might mean that early non-sepsis would not be found in time. Unfortunately, the clinical signs and symptoms of neonatal infection can often be occult and can easily evolve into sepsis. Studies have shown that PCT begins to increase early in an infection, within 4 hours, followed by a rise in CRP^31,32^. The present study found that there was a significant difference in PCT between the sepsis group and the non-sepsis and control groups, but unfortunately, there was no significant difference between the non-sepsis group and the control group. We thought this might be due to the characteristics of the newborn themselves or the small number of patients included in this study, leading to the lack of difference between the non-sepsis group and the control group. Further research is needed in the future. WBC and CRP increase slowly after bacterial infection or inflammatory reactions occur, which makes it easy to delay the diagnosis time and allow the infection to worsen. In this study, there were significant differences in the WBC and CRP levels between the sepsis and control groups, and there were no significant differences between the non-sepsis group and the control or sepsis group, which is consistent with the clinical situation.

Early studies had shown that CD64 molecules can be used as biomarkers for the diagnosis of bacterial infections^7,33^. Our result showed that the CD64 indices of the sepsis and non-sepsis groups were significantly higher than those of the control group. Therefore, it provided evidence for the early and rapid diagnosis of neonatal infections and of the value of the CD64 index in clinical practice. However, there was no difference in the CD64 index between the sepsis group and the non-sepsis group. This might be because the expression levels of CD64 in the neutrophils were not elevated over a longer period. A study had also shown that in the early stages of infection, CD64 gradually increased on the surface of granulocytes, which could be maintained for several days upon reaching a certain level^34^. Another interesting finding was that in the sepsis group, PCT was significantly increased compared with that in the non-sepsis group. Thus, we thought PCT might be an important indicator to determine whether a child was progressing from non-sepsis to sepsis. Meanwhile, in the sepsis group, the CD64 index was positively correlated with CRP. Because the sensitivity and specificity of CRP for the diagnosis of neonatal infections were not satisfactory, the CD64 index might be a viable alternative to CRP.

In this study, NLR was significantly different between the sepsis group and the control group, and NLR was positively correlated with WBC in the sepsis group. This indicated that more neutrophils were involved in the immune response for more-serious cases of sepsis. Additionally, to our surprise, the NLR in the sepsis group was negatively correlated with the CD64 index. It is possible that in sepsis, mature neutrophils are consumed in large quantities, and the body therefore releases immature neutrophils to further defend against the bacterial invasion. These neutrophils are not fully activated and express minimal to no CD64. The greater the number of immature neutrophils, the lower the total MFI of CD64 was on these cells. This may be why the CD64 index was not different between the non-sepsis group and the sepsis group.

By comparing the ROC curves of CD64 and the traditional infection indices in the infected group, we found that the AUC of the CD64 index was greater than that of the WBC and CRP. We expected that the CD64 index would have a more significant advantage in the diagnosis of infection than the WBC and CRP. Nevertheless, the AUC of the CD64 index was slightly smaller than that of the PCT, and both had good sensitivity and specificity. Overall, there were many similarities and some complimentary aspects between the CD64 index and PCT in the diagnosis and treatment of neonatal infection.

In conclusion, the CD64 index could play an ancillary role in the diagnosis and treatment of neonatal infection, as it is a more reliable index for early diagnosis than WBC, CRP and PCT. However, compared with the CD64, WBC and CRP indices, the PCT clearly has more advantages and diagnostic value during late infection (at the phase of sepsis). Therefore, the combined assay of the CD64 index and other traditional experimental indicators of infection, especially PCT, can enhance the diagnostic accuracy for neonatal infections in the clinic.

## References

[1] El Shimi MS, Abou Shady NM, Hamed GM, Shedeed NS (2017). Significance of neutrophilic CD64 as an early marker for detection of neonatal sepsis and prediction of disease outcome. J. Matern. Fetal. Neonatal. Med..

[2] Lawn JE (2014). Every newborn: progress, priorities, and potential beyond survival. Lancet.

[3] Nour I (2017). Risk factors and clinical outcomes for carbapenem-resistant Gram-negative late-onset sepsis in a neonatal intensive care unit. J. Hosp. Infect..

[4] Pradhan R (2016). Ratio of neutrophilic CD64 and monocytic HLA-DR: A novel parameter in diagnosis and prognostication of neonatal sepsis. Cytometry B Clin. Cytom..

[5] Paolucci M, Landini MP, Sambri V (2012). How can the microbiologist help in diagnosing neonatal sepsis?. Int. J. Pediatr..

[6] ten Oever J, Netea MG, Kullberg BJ (2016). Utility of immune response-derived biomarkers in the differential diagnosis of inflammatory disorders. J. Infect..

[7] Papadimitriou-Olivgeris M (2015). Role of CD64 expression on neutrophils in the diagnosis of sepsis and the prediction of mortality in adult critically ill patients. Diagn. Microbiol. Infect. Dis..

[8] Delanghe JR, Speeckaert MM (2015). Translational research and biomarkers in neonatal sepsis. Clin. Chim. Acta.

[9] Kipfmueller F (2015). Role of neutrophil CD64 index as a screening marker for late-onset sepsis in very low birth weight infants. PLoS One.

[10] Franz AR (2004). Measurement of interleukin 8 in combination with C-reactive protein reduced unnecessary antibiotic therapy in newborn infants: a multicenter, randomized, controlled trial. Pediatrics.

[11] Miyake F (2016). Analysis of the physiological variation in neutrophil CD64 expression during the early neonatal period. Am. J. Perinatol..

[12] Bhandari V (2014). Effective biomarkers for diagnosis of neonatal sepsis. J. Pediatr. Infect. Dis. Soc..

[13] Mussap M, Noto A, Cibecchini F, Fanos V (2013). The importance of biomarkers in neonatology. Semin. Fetal Neonat. Med..

[14] Turner D (2006). Procalcitonin in preterm infants during the first few days of life: introducing an age related nomogram. Arch. Dis. Child. Fetal Neonatal Ed..

[15] Simonsen KA, Anderson-Berry AL, Delair SF, Davies HD (2014). Early-onset neonatal sepsis. Clin. Microbiol. Rev..

[16] Lynema S, Marmer D, Hall ES, Meinzen-Derr J, Kingma PS (2015). Neutrophil CD64 as a diagnostic marker of sepsis: impact on neonatal care. Am. J. Perinatol..

[17] Altunhan H, Annagür A, Örs R, Mehmetoğlu I (2011). Procalcitonin measurement at 24 hours of age may be helpful in the prompt diagnosis of early-onset neonatal sepsis. Int. J. Infect. Dis..

[18] Stocker M, Hop WC, van Rossum AM (2010). Neonatal procalcitonin Intervention Study (NeoPInS): effect of procalcitonin-guided decision making on duration of antibiotic therapy in suspected neonatal early-onset sepsis: a multi-centre randomized superiority and non-inferiority Intervention Study. BMC Pediatr..

[19] Huizinga TW, Van der Schoot CE, Roos D, Weening RS (1991). Induction of neutrophil FC-gamma receptor I expression can be used as a marker for biologic activity of recombinant interferon-gamma *in vivo*. Blood.

[20] Fjaertoft G, Håkansson L, Ewald U, Foucard T, Venge P (1999). Neutrophils from term and preterm newborn infants express the high affinity Fcgamma-receptor I (CD64) during bacterial infections. Pediatr. Res..

[21] Hoffmann JJ (2011). Neutrophil CD64 as a sepsis biomarker. Biochem. Med. (Zagreb).

[22] Hoffmann JJ (2009). Neutrophil CD64: a diagnostic marker for infection and sepsis. Clin. Chem. Lab. Med..

[23] Choo YK, Cho HS, Seo IB, Lee HS (2012). Comparison of the accuracy of neutrophil CD64 and C-reactive protein as a single test for the early detection of neonatal sepsis. Korean J. Pediatr..

[24] Nupponen I (2002). Neutrophil activation in preterm infants who have respiratory distress syndrome. Pediatrics.

[25] Dilli D, Oğuz ŞS, Dilmen U, Köker MY, Kızılgün M (2010). Predictive values of neutrophil CD64 expression compared with interleukin-6 and C-reactive protein in early diagnosis of neonatal sepsis. J. Clin. Lab. Anal..

[26] Chang, L. W. Neonatal infectious diseases in *Pediatrics*, 8th ed. (ed. Wang, W. P.) 127–128 (China, People’s Medical Publishing House Co. 2013).

[27] Soni S (2013). Evaluation of CD64 expression on neutrophils as an early indicator of neonatal sepsis. Pediatr. Infect. Dis. J..

[28] Schuetz P (2015). Economic evaluation of procalcitonin-guided antibiotic therapy in acute respiratory infections: a US health system perspective. Clin. Chem. Lab. Med..

[29] Lam HS (2013). Neutrophil CD64 for daily surveillance of systemic infection and necrotizing enterocolitis in preterm infants. Clin. Chem..

[30] Polin RA (2012). Management of neonates with suspected or proven early-onset bacterial sepsis. Pediatrics.

[31] Zhydkov A (2015). Utility of procalcitonin, C-reactive protein and white blood cells alone and in combination for the prediction of clinical outcomes in community-acquired pneumonia. Clin. Chem. Lab. Med..

[32] Ng PC, Lam HS (2010). Biomarkers for late-onset neonatal sepsis: cytokines and beyond. Clin. Perinatol..

[33] de Jong E (2016). Neutrophil CD64 expression as a longitudinal biomarker for severe disease and acute infection in critically ill patients. Int. J. Lab. Hematol..

[34] Layseca-Espinosa E (2002). Expression of CD64 as a potential marker of neonatal sepsis. Pediatr. Allergy Immunol..

